# Imaging modalities for endoleak surveillance

**DOI:** 10.1002/jmrs.522

**Published:** 2021-06-18

**Authors:** Aman Berry Williams, Zoheb Berry Williams

**Affiliations:** ^1^ Department of Vascular Surgery Gold Coast University Hospital Southport Queensland Australia

**Keywords:** Aortic aneurysm, diagnostic imaging, endoleak, endovascular procedures, vascular grafting

## Abstract

As the global population ages, the issue of abdominal aortic aneurysm continues to grow. With the evolution of new devices and refined operative technique, aneurysm treatment via endovascular aortic repair is becoming increasingly favourable. This, however, is not without drawbacks, where regular surveillance is paramount to long‐term success and detection of post‐procedure complications. Of these complications, endoleak is the most notable and poses the greatest risk of potential future aortic rupture. The purpose of this review paper is to discuss the armada of imaging modalities used in the detection and evaluation of endoleak and their varying usefulness. Plain abdominal X‐ray is a cost‐effective tool in detecting gross graft abnormalities such as stent migration or deformity (kinking or fracture). Though it may raise suspicion for endoleak, X‐ray does not allow accurate classification of endoleak type when used alone. Duplex ultrasonography quantifies both aortic anatomy and real time flow dynamics. Most screening programmes are conducted using two‐dimensional ultrasound. Unfortunately, observer and equipment variability may lead to surveillance discrepancies—but reduced when utilising a dedicated vascular sonography laboratory. Contrast enhanced ultrasonography is a promising alternative to computed tomography, though still is emerging. Computed tomography angiography certainly has disadvantages (ionising radiation, contrast‐nephropathy, limited differentiation of endoleak type)—however, it provides near‐real surgical dimensions and highlights graft complications and concomitant disease (such as neighbouring infection). With widespread availability and short scan time, it certainly remains valuable in surveillance. Magnetic resonance angiography has a similar sensitivity to computed tomography (minus the radiation), however is plagued by movement and metal artefact. Other novel modalities in endoleak surveillance include four‐dimensional ultrasound, multiplanar intra‐operative probes, nuclear medicine and wall stress analysis.

## Graphical abstract

As the global population ages, the issue of abdominal aortic aneurysm continues to grow. Endovascular aortic repair has helped stem this issue, but from it arises the challenge of managing endoleak. We review the armada of imaging modalities available for the detection and evaluation of endoleak.

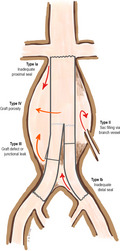

## Introduction

It is well documented that the prevalence of abdominal aortic aneurysm (AAA) certainly increases per decade of life and is additionally four to six times more common in men.^1^, ^2^ An Australian study quotes a prevalence of 7.2% for aneurysms >30 mm in transverse diameter in men between the ages of 65 and 83.^3^ Consideration for operative intervention is somewhat governed by the patient’s age and pre‐morbid physiologic baseline. A rapidly evolving treatment technology, the endovascular aortic aneurysm repair (or EVAR), is becoming increasingly favourable and may also be opening the door to treating increasingly elderly and comorbid patients.^4^

A disadvantage, however, of the endovascular technique is the need for regular monitoring and strictly orchestrated follow‐up. This is the case given the potential immediate and delayed procedure specific complications of endovascular repair, including endoleak, aneurysm rupture, graft limb occlusion and graft migration. These complications account for a reintervention rate that increases by as much as 11–12% each year for at least the first three post‐operative years.^5^ Another study quotes a reintervention rate of 10% after EVAR but with 92% of the reinterventions using endovascular techniques. There are fewer complications and reinterventions with later generation devices, but it is difficult to determine if this phenomenon offsets the likely net increase in reinterventions as a result of a greater proportion of AAA’s being treated endoluminally. This review will focus on the surveillance of endoleak post‐EVAR alone, with discussion pertaining to their usefulness.

**Figure 1:**
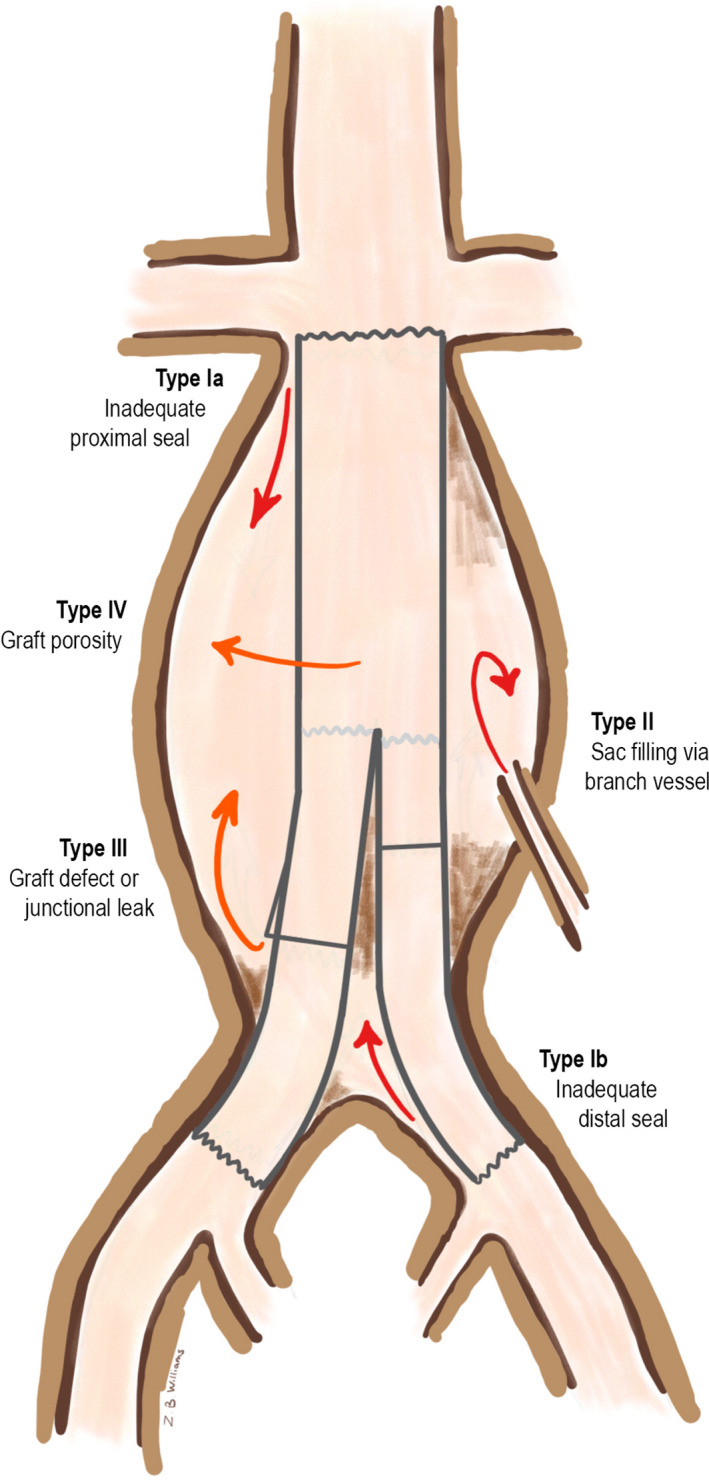
Anatomical classification of endoleak.

There are several imaging modalities available for post‐EVAR surveillance, some of which include plain film radiographs, duplex and colour duplex ultrasonography (DUS or CDUS), contrast enhanced sonography (CEUS), computed tomographic angiography (CTA) and magnetic resonance angiography (MRA). This brief review aims to discuss them in regard to their role in endoleak detection and surveillance only. Endoleaks are classified anatomically, based on the location of the leak.^6^, ^7^ This can be seen demonstrated in Figure 1 below. Arterial blood leaking into the aneurysm sac comprises a type I endoleak, this type is stratified into leaks occurring from the proximal (Ia) and distal (Ib) attachment sites. Type II endoleaks arise from collateral vessels communicating with the aneurysm sac. While these are the most common type of endoleak, frequent spontaneous thrombosis and bidirectional flow make the treatment of these endoleaks a point of discussion and controversy.^7^ A type III leak may be due to a junctional failure, a midgraft puncture or other graft failures. Type IV endoleaks are rare and almost exclusively observed at the time of the first post‐graft angiogram; they are due to porosity of the material of the graft, a problem faced particularly with earlier generation endografts. Finally, type V endoleaks are due to endotension, and this is largely a diagnosis of exclusion.

## Plain Abdominal X‐ray

Plain abdominal radiography is a simple and cost‐effective adjunctive tool in the surveillance of EVAR patients, provided patient‐positioning and image capture techniques are protocol driven.^8^ Though it cannot directly be used to detect endoleak, it certainly can raise the suspicion of, or give clues to the reason for endoleak. For a minimal radiation burden, the test can yield important and accurate information on graft component migration and any stent deformities (such as kinking and fracture).^9^ Unlike CTA, MRI and DUS, plain radiography does not suffer so severely from artifacts,^7^ but it is understandably inaccurate for the classification of endoleak. Obvious stent defects found on a plain radiograph should always prompt further delineation with DUS or CTA.

## Duplex Ultrasonography

Colour duplex ultrasonography (CDUS) combines standard brightness‐mode (B‐mode) imaging with Doppler ultrasound and a colour overlay to provide information on the anatomical features of the aorta and the stent‐graft, as well as dynamic flow information in real time. Repair guidelines and most population screening programmes are currently based on 2D CDUS.^3^, ^10^, ^11^ Three‐dimensional CDUS is an emerging technology which creates a dynamic reconstruction, from images obtained by a multiplanar probe, with specialised software that allows the view to manipulate images in a similar manner to CTA imaging.^12^

Ultrasound has been rivalled with CTA, which has long been considered the gold standard for EVAR surveillance, extensively in the medical literature. Compared with CTA, DUS appears to be troubled by a high inter‐ and occasionally intra‐observer variability;^7^, ^13^ this may be related to the observers or to differences in equipment.^14^ But some recent studies have unintentionally demonstrated that by using a dedicated vascular sonography laboratory with trained staff and a strict protocol, consistently, there is significant agreement.^15^, ^16^

Compared with protocol‐based CTA, CDUS consistently has a lower sensitivity. A meta‐analysis of 31 studies suggested a sensitivity of 74%,^17^ while other studies describe a sensitivity between 62% and 83%.^14^ Interestingly, the sensitivity of CDUS has been reported as high as 100% in a study using an accredited vascular sonographer and a standard scanning protocol.^15^ Most CTA’s are performed with a non‐contrast and an arterial contrast phase protocol, with many standard follow‐up protocols also including a delayed phase. When CDUS is compared with CTA that does not routinely use a triple‐phase contrast protocol, it may perform superiorly for the detection of endoleaks,^18^ and there are mounting concerns about the radiation exposure involved in routine triple‐phase CTA for EVAR surveillance.

While CDUS has a positive predictive value as low as 42%,^19^ it consistently performs with a high specificity, approaching 99%^7^, ^9^, ^12^, ^14^, ^16^, ^20^ and a negative predictive around 94–100%.^15^, ^19^

Colour duplex ultrasonography deserves a spotlight for several other reasons. In the subset of type I and type III endoleaks, CDUS has a sensitivity and specificity of 83% and 100%, respectively.^17^ It is conceivable that low‐flow type II endoleaks may be missed on CTA and there is emerging evidence that CDUS outperforms CTA for the detection of this specific type of endoleak.^19^

Surveillance is important for identifying the need for reintervention. It is generally agreed that type I and type III endoleaks warrant early intervention but there is no consensus on the treatment of type II endoleaks, unless aneurysm sac expansion is noted over time. DUS has been shown to be equivalent to CTA for measuring aneurysm size and detecting sac expansion.^20^ A recent retrospective study has also suggested that DUS outperforms CTA for the detection of endoleak requiring intervention – DUS identified 89% of the patients that went on to need intervention for their endoleak, while CTA only detected 58% but this research does come with all the caveats of retrospective design.^19^

Beyond the numbers, CDUS has a number of limitations. The quality of the image is always potentially compromised by obesity, arterial wall calcification, bowel gas, ascites, abdominal wall hernias and subcutaneous emphysema.^12^ Some patients may need to be placed on low residue diets or fasted for the scan to provide enough information. The scan is also time intensive, where the training and experience of the observer and the quality of the equipment may significantly impact the study.^13^ Despite this, DUS is relatively cheap, very portable and readily accessible.^13^ Additionally, there is no radiation involved and the threats of nephrotoxicity or anaphylaxis from contrast allergy are non‐existent. This does make it an appealing modality for EVAR surveillance.

## Contrast‐Enhanced Ultrasonography

Contrast‐enhanced ultrasonography (CEUS) works the same way as standard colour duplex ultrasonography but employs the use of an intravenous contrast agent. Current approved agents include perfluorocarbon and sulphur hexafluoride which are administered as microbubbles.^12^, ^14^ Each microbubble is surrounded by a phospholipid outer which appears as an echo‐reflection during the scan. The standard dosing is between 1 and 2.4 mL per bolus, with better image quality seen using higher doses.^14^, ^16^ Boluses can be repeated and in fact often have to be because of the limited field of view the ultrasound probe provide per pass of contrast.^21^ This unfortunately does mean that the scan time is comparable to that of CDUS or even slightly longer.

The contrast agent itself has an extremely low adverse effect profile. The microbubbles gradually embolize to the lungs where they are destroyed and it is safe for use in renal impairment.^22^ Contrast nephropathy has not been reported, there is no extra radiation involved and the risk of life threatening anaphylaxis is 0.001%.^22^ Despite a warning to avoid using these agents in patients with recent acute coronary syndrome,^14^ overall it appears that the enhancement of DUS with contrast is quite safe.

Like DUS, CEUS has been compared with CTA in the medical literature as a potential replacement for CTA surveillance. CEUS has a reported sensitivity of 93% to 99% and a specificity of 100% for identifying endoleak post‐EVAR.^17^, ^23^ While this is quite promising, CEUS will have a poorer performance with some grafts that have a higher tendency to produce echo‐reflection artefacts early after implantation, like those crafted with expanded polytetraflouroethylene which tend to have this property for their first 6 months.^24^

Combining DUS with an intravenous contrast appears to increase the sensitivity of the test and decrease the inter‐observer variability, particularly when combined with 3D rendering software and multiplanar probes.^12^ The performance of CEUS has been so promising that many reviewers are advocating for CEUS as an alternative to CTA^24^, ^25^, ^26^, ^27^ for the surveillance of EVAR patients. Compared with its non‐contrast equivalent, CEUS does not perform significantly better for type I and III endoleaks specifically – the sensitivity of DUS is 83% with a 95% confidence interval of 40–97% in one meta‐analysis, the same study quotes a sensitivity of 99% for CEUS but a confidence interval of 25–100% casts doubt about a true improvement in sensitivity in this subgroup of endoleaks.^17^ Regardless, CEUS has repeatedly been shown to be more accurate for the classification of endoleaks and more sensitive for the detection of type II endoleaks than CTA.^28^, ^29^ It probably outperforms DUS in detecting low‐flow type II endoleaks but more comparisons need to be made to be sure.^16^

This imaging modality is limited by the same patient factors as DUS and is less accessible and slightly more expensive because it is a specialised test and contrast administration requires personnel, training and protocol change. While a lot of the literature on CEUS is emerging from Europe, CEUS is not routinely being used for EVAR surveillance in Australia, to our knowledge. It does still remain a promising alternative to CTA and does provide flow dynamic information that CTA does not.

## Computed Tomography Angiography

Computed tomography has developed rapidly over the past decade, and newer machines with greater precision are slowly improving the industry by lowering the radiation doses patients need to be exposed to. The radiation dose from a CTA may be enough to provoke cancer in as many as 1 in 2000 people,^9^ with the digestive organs receiving the majority of the radiation.^30^ Extending the radiation dose to cover a triple‐phase CTA (allowing detection of low flow endoleak), the risks may be even higher. Current European guidelines suggest follow‐up at 1, 6 and 12 months after EVAR and then annually thereafter.^9^, ^12^, ^16^ This totals a significant radiation burden for the individual EVAR patient. Outside of Europe, CTA is often the primary imaging modality for surveillance, because of its availability and its use in several major trials such as the EUROSTAR and UK EVAR trials.^14^

Beyond the radiation dose, the scan generally requires the use of an iodine‐based contrast. Though current literature suggests that there is no risk to renal function for intravenous iodinated contrast, the anecdotal experience of nephropathies post‐contrast remains a strong perception in the endovascular surgical community. Despite saline and sodium bicarbonate pre‐hydration protocols, post‐CTA nephropathy may affect up to 12% of patients receiving the scan, and 7% in those with no prior predisposing kidney disease.^9^, ^20^, ^31^ Ultimately given these concerns, other imaging modalities should be strongly considered in the setting of renal impairment.

Despite these drawbacks, CTA does have a number of valuable qualities which have certainly contributed to its popularity for EVAR surveillance. Of all the available imaging modalities, CTA provides images and aortic dimensions closest to real surgical specimens. Other modalities particularly standard DUS tend to slightly underestimate the aortic diameter.^12^ It is also a good imaging modality for investigating other potentially concurrent issues, including soft tissue defects such as abscesses, infections and inflammatory exudate.^7^

The scan time itself is relatively short – an initial non‐contrast phase can be done rapidly, an arterial phase is generally performed 30 sec after injection of intravenous contrast, and delayed phases occur at between 80 and 300 sec post‐contrast.^14^ The majority of time may actually be spent in the processing of the image. The spatial resolution afforded by modern CTA is currently unparalleled by any other non‐invasive imaging modality and, unlike ultrasound, it can characterise important graft complications such as fracture, kinking or migration.^7^ Duplex needs to be combined with protocol‐based abdominal radiographs to be able to do the same.

In a meta‐analysis, with CEUS as the comparator, CTA performed with a sensitivity of 70% and a specificity of 98%.^17^ When type I and III endoleaks are separated in their own subset analysis, this improves to a sensitivity and specificity of 93% and 100%, respectively.^17^ This fairly accurately describes the major limitation of CTA – despite advances in CTA imaging and processing technology, a portion of type II endoleaks are being consistently missed. This phenomenon has been supported in the literature in a number of individual studies and reviews.^9^, ^13^, ^15^, ^20^ Further, CTA is less accurate in classifying the type of endoleak than duplex or CEUS.^19^, ^29^ It is unclear whether the type of CTA protocol has an impact on the accuracy of the CTA in classifying the type of endoleak. Some literature suggests that arterial phase scanning (without a delayed phase) may not significantly reduce the sensitivity of CTA.^32^ The current understanding is that given endoleak is generally a low‐pressure phenomenon, unless the CT phase is sufficiently delayed the leak may not be seen – alternatively the longer duration ultrasound scan circumvents this issue.

The cost of one CTA scan has been cautiously estimated at around 500 Euro.^15^ Obviously, this is highly variable and will change between departments, between imaging providers, by the age and quality of the scanning machine and its ongoing running and maintenance costs – and of course, the price may increase or decrease over time and vary with the level of government and corporate subsidisation – as with the other modalities. In a 2012 study of 235 CT scans which had comparable CDUS scans from the same patients, CDUS did not miss any endoleaks that were detected by CTA. The authors subsequently suggested that it would be reasonable to have replaced some CTA scans with CDUS alone, without compromising accuracy, such that the total number of CTA scans falls to 36 for this cohort, making a saving in excess of 82,000 euro per year.^15^ Others suggest that more than 65% of the post‐operative costs associated with EVAR are due to CT scanning alone.^33^ In U.S. dollars, a potential saving of $1595 per patient per year (excluding medicare benefits) could be seen by replacing CTA surveillance with DUS^20^ – this statistic does not appear to take into account the cost of adding radiography to monitor macroscopic graft complications.

Based on this information, some authors advocate for a limited non‐contrast CT to identify sac enlargement, and given a then high suspicion for endoleak, proceed to a contrast enhanced CT scan.^7^ Others advocate for CTA to be replaced entirely by CEUS,^14^ and yet another group suggest that the current early screening is too frequent and that a 6‐month CTA can be avoided if scans at 1 month appear normal.^9^, ^34^ Nevertheless, CTA is still not easily replaced, perhaps because of its availability, high spatial resolution, its use as a planning tool and because of readily available software packages which allow rapid manipulation of images. Additionally, modalities such as CEUS are not widely available and have a more difficult learning curve for performance and for interpretation.

## Magnetic Resonance Angiography

Like CTA, MRA is generally performed in multiple stages. Most protocols begin with an axial T1‐weighted gradient echo image, followed by phases before and after intravenous gadolinium administration. MRA has a similar sensitivity to CTA and even outperforms it regarding the detection of type II endoleaks in nitinol EVARs specifically^7^, ^14^ and a recent review suggested both a sensitivity and specificity of 100%.^23^ Still, it has several limitations, which prevent its widespread use.

This imaging modality is perhaps plagued the most by movement and metal artefact out of all the modalities discussed so far. While nitinol stent grafts and platinum coils are easily visualised and cause little artefactual disturbance, elgiloy (an alloy of nickel, cobalt and chromium) and nickel alloy stents cause blurring from movement, and stainless steel can cause significant artefact and may even fracture or dislocate during the scan.^7^, ^9^ Despite a lack of ionising radiation and no direct nephrotoxic effects related to the contrast agent, gadolinium may be associated with nephrogenic systemic fibrosis which at this stage appears to be a condition unique to renally impaired patients.^7^ Gadolinium does, however, carry a lower risk of nephrotoxic reaction when compared to iodinated contrast media.^14^ MRA is also dearer than CTA, with greater purchase and maintenance costs and has a longer procedure time. Finally, it may not be suitable for claustrophobic patients.^14^

## Novel Modalities and Nuclear Medicine

Three‐dimensional DUS and CEUS are emerging as useful and accurate imaging modalities. They may well be an acceptable replacement for CTA in the close future and would minimise the burden of contrast complications and radiation while potentially decreasing the cost of surveillance. These technologies are not available everywhere and will have a learning curve, so any transition from CTA will have to be a slow one. Four‐dimensional modalities have also been described, but this description relates to those modalities being able to produce three‐dimensional images that are dynamic and can be viewed across an interval of time.^12^ Multiplanar probes are being used intra‐operatively to identify endoleaks which may not even be seen on intra‐operative angiography, and 3D reconstruction technology is helping to reduce the inter‐observer variability seen with ultrasound.^12^, ^35^ Newer software platforms now enable CEUS to be anchored to a previous or calibrating CTA or MRA image to assess or improve its accuracy^14^ – but this method is perhaps more academic than it is useful.

Regarding nuclear medical imaging modalities, there is limited evidence that they can be accurately and effectively used in detecting and classifying endoleaks after EVAR, and some evidence suggesting that their accuracy is not yet comparable with CTA.^36^ In the presence of enlarging sac size post‐EVAR, and the absence of endoleak detection using the aforementioned routine surveillance imaging modalities, nuclear medicine does have some utility in assessing further for potential infective and inflammatory processes contributing to ongoing AAA enlargement – though this is an entirely different pathology to endoleak.

Aneurysm size does not appear to be the sole indicator of rupture risk. Many large aneurysms do not rupture, and some small ones do. The fate of the large or growing remaining sac post‐EVAR has not been studied for obvious ethical concerns, where an aneurysm with a endoleak confers a risk of ongoing sac pressurisation and therefore AAA rupture risk. With this in mind, it is conceivable that surveillance modalities will target different endpoints in the future. Wall stress is one such candidate, and wall stress analysis is now possible (in a limited capacity) with CT scanning – where high‐resolution images can detect intramural stranding and local wall movement defects.^12^ This was previously not possible with standard DUS, but 3D reconstructions and contrast‐enhancement is making even this possible.^37^ This type of information might be particularly useful in the case of type II endoleaks, particularly those with stable or minimally growing aneurysm sacs.

## Conclusion

Innovation has led to an armada of endoleak surveillance imaging tools, where the global literature pits one modality against another, and describes a number of modalities that simply are not available everywhere. Regardless, surveillance plans need to be tailored to the individual patient. Endoleaks will likely continue to be a problem until there are significant changes or advances in stent‐graft technology. The timing intervals appear to be largely agreed on, with surveillance scans planned at 1 and 12 months, and often another scan at 6 months.^38^, ^39^ Current mainstream modalities for endoleak surveillance include ultrasound and CTA, and however, this will expectantly evolve as efficiency, sensitivity and specificity improves. Ultimately, the imaging modality of choice will depend not just on the information above, but the availability of each modality in the patient’s locale and the preference of the clinician.
